# Long-term endoscopic gastric mucosal changes up to 20 years after *Helicobacter pylori* eradication therapy

**DOI:** 10.1038/s41598-024-63928-6

**Published:** 2024-06-06

**Authors:** Eri Iwata, Mitsushige Sugimoto, Yoshika Akimoto, Mariko Hamada, Ryota Niikura, Naoyoshi Nagata, Kyosuke Yanagisawa, Takao Itoi, Takashi Kawai

**Affiliations:** 1 Department of Gastroenterological Endoscopy, Tokyo Medical University Hospital, 6-7-1 Nishishinjuku, Shinjuku-ku, Tokyo 160-0023 Japan; 2 Division of Genome-Wide Infectious Microbiology, Research Center for GLOBAL and LOCAL Infectious Disease, Oita University, 1-1 Idaigaoka, Hasama, Yufu, Oita 879-5593 Japan; 3 Department of Gastroenterology and Hepatology, Tokyo Medical University Hospital, 6-7-1 Nishishinjuku, Shinjyuku-ku, Tokyo 160-0023 Japan

**Keywords:** *Helicobacter pylori*, Eradication therapy, Atrophy, Intestinal metaplasia, Map-like redness, Gastric cancer, Gastric cancer, Cancer

## Abstract

*Helicobacter pylori* eradication therapy reduces the risk of gastric cancer. However, it is unclear whether the severity of risk factors for gastric cancer such as atrophy and intestinal metaplasia are reduced after eradication in the long term. We aimed to study long-term changes in endoscopic risk factors for gastric cancer up to 20 years post-eradication. The endoscopic severity of gastritis according to the Kyoto Classification of Gastritis in 167 patients was retrospectively evaluated over an average follow-up 15.7 years. A significant improvement in mean total gastric cancer risk score (4.36 ± 1.66 to 2.69 ± 1.07, *p* < 0.001), atrophy (1.73 ± 0.44 to 1.61 ± 0.49, *p* = 0.004), and diffuse redness (1.22 ± 0.79 to 0.02 ± 0.13, *p* < 0.001) was observed compared to baseline in the Eradication group. However, there was no change in the never infection and current infection groups. The frequency of map-like redness increased over time until 15 years (3.6% to 18.7%, *p* = 0.03). The Cancer group had significantly higher risk scores at all time points. Endoscopic atrophy significantly improved in eradicated patients over long-term, suggested that eradication is one of the key elements in gastric cancer prevention. Individualized surveillance strategies based on endoscopic gastritis severity before eradication may be important for those at risk of gastric cancer.

## Introduction

*Helicobacter pylori* infection is a major risk factor for gastric cancer, and is also known as a cause of upper gastrointestinal diseases, such as peptic ulcer, atrophic gastritis, gastric adenoma, and gastric mucosa-associated lymphoid tissue lymphoma^1,2^. *H. pylori* eradication therapy is known to be effective in preventing the onset of *H. pylori*-associated diseases, and the International Clinical Guidelines for *H. pylori* Infection recommend that *H. pylori*-infected patients receive eradication therapy as the first-line treatment^3–5^.

Although eradication therapy has been shown to prevent the onset of gastric cancer, the incidence of metachronous cancer after eradication therapy is about 0.3% per year, indicating that eradication therapy cannot completely prevent the development of gastric cancer^6,7^. Therefore, to not overlook gastric cancer in patients currently infected with *H. pylori* and in those who previously received eradication therapy, it is important to stratify each individual’s risk based on risk factors for gastric cancer development. In general, risk factors for gastric cancer include environmental factors (e.g., *H. pylori* infection, salt intake, smoking, and consumption of red meat), host genetic factors (e.g., family history, Lynch syndrome, and autoimmune gastritis), and bacterial factors (e.g., *H. pylori* virulence factors). In addition, endoscopic and pathological risk factors include gastric atrophy, intestinal metaplasia, fold enlargement, nodularity, and diffuse redness^8–10^. As an evaluation method of atrophy and intestinal metaplasia, in Europe and the United States, a scoring system based on histopathological evaluation methods of the gastric mucosa (Updated Sydney System, OLGA classification, and OLGIM classification) is being used^11–13^. However, in Japan, the usefulness of risk assessment based on the Kyoto Classification of Gastritis using endoscopic findings has been reported^9,14–16^. Together with such endoscopic findings, it is important to identify additional risk factors, such as other clinical findings and symptoms, and establish a screening method that stratifies post-eradication gastric cancer risk.

Recently, it has been reported that time-dependent changes in the severity and status of endoscopic risk factors for gastric cancer occur after *H. pylori* eradication^17–19^. These time-dependent changes of risk factors and severity may change an individual’s risk of gastric cancer. Therefore, as well as stratifying the risk of gastric cancer at the time of eradication, it is considered necessary to carefully monitor the progression and changes in gastric cancer risk of a patient by long-term endoscopic surveillance. Although several reports evaluating changes in atrophy and intestinal metaplasia in *H. pylori* eradicated patients are available^17–19^, studies evaluating long-term changes are limited. In addition, because histological evaluation by biopsies cannot be performed in all patients, owing to its cost and risk of post-biopsy bleeding, we believe that an endoscopic evaluation system will be useful for risk assessment of gastric cancer after eradication in actual examinations.

Therefore, we conducted this study to identify endoscopic risk factors for gastric cancer after *H. pylori* eradication, by obtaining information on long-term endoscopic changes of *H. pylori-*infected patients with gastritis after eradication.

## Methods

### Patients and study design

This study was a single-center, retrospective observational study. Patients who had undergone regular endoscopic examinations for health check-ups for 10 years or more at Tokyo Medical University Hospital were included, and their endoscopic findings at the first endoscopy, as well as findings 5 years, 10 years, 15 years, and 20 years subsequently were retrospectively reviewed. The patient’s *H. pylori*-infection status and changes in endoscopic findings, such as gastric atrophy and intestinal metaplasia, and their severity according to the Kyoto Classification of Gastritis were evaluated^8^. The inclusion criteria were being 20 years or older, and having clear endoscopic images for the evaluation of gastric condition. Patients in whom the upper gastrointestinal tract (esophagus and stomach) was surgically removed during follow-up, patients in whom the follow-up period was less than 10 years, and patients in whom it was difficult to evaluate the status of *H. pylori* infection using medical records were excluded. The history of *H. pylori* eradication, lifestyle history of alcohol and smoking, family history of cancer, and past medical history were analyzed.

Patients were categorized into 3 groups, as follows: the Eradication group, the Current infection group, and the Never infection group. The Eradication group was defined as patients who underwent eradication treatment during the follow-up period, and patients who had already received eradication therapy at the time of the first endoscopy. The Current infection group was defined as patients who had not undergone eradication during the follow-up period, and the Never infection group was defined as patients with no endoscopic, pathological, or serological signs of *H. pylori* infection.

The study protocol adhered to the ethics principles of the Declaration of Helsinki, and was approved by the institutional review board of Tokyo Medical University (study approval no.: T2021-0265). Because this study was conducted using a retrospective design, written informed consent was not obtained from each enrolled patient.

### Endoscopic examination

Endoscopic examinations were performed using an Olympus electroscope (GIF-N230, GIF-N260, GIF-XP260N, GIF-XP290N, GIF-1200N, and others; Tokyo, Japan) depending on the timing. Five endoscopic findings (atrophy, intestinal metaplasia, fold enlargement, nodularity, and diffuse redness) of the gastric cancer risk score were scored according to the Kyoto Classification of Gastritis, and the severity of atrophy according to the Kimura-Takemoto classification. In addition, we evaluated map-like redness and hiatal hernia. The 5 endoscopic findings of the gastric cancer risk were scored as follows. (1) Atrophy: Atrophy was defined a visible capillary network, low niveau, and yellowish pale in color as atrophic features, while diffuse redness with high mucosal height as characteristics of non-atrophy^20^. Endoscopically graded mucosal atrophy was scored as 0 (C-0, C-1), 1 (C-2, C-3), and 2 (O-1, O-2, and O-3) according to the Kimura-Takemoto classification^20^. (2) Intestinal metaplasia endoscopically observes as grayish-white elevated lesions forming an irregular uneven surface. Useful endoscopic findings for diagnosis of intestinal metaplasia include a whitish-colored villous pattern and rough surface^21,22^. Intestinal metaplasia was scored as 0 (none), 1 (antrum), and 2 (corpus). (3) Fold enlargement: An enlarged fold observed in the corpus is defined as a crawling fold enlargement with a width ≥ 5 mm that is only partially flattened or is not flattened by sufficient gastric insufflation. Hypertrophic fold enlargement was scored as 0 (< 5-mm width) and 1 (≥ 5-mm width). (4) Nodularity: nodular gastritis, which is defined as a small granulated pattern in the antrum, was scored as 0 (absence) and 1 (presence). (5) Diffuse redness: diffuse redness is defined as uniform redness of the corpus mucosa^23^. No diffuse redness is scored as score 0, mild diffuse redness or regular arrangement of collecting venules (RACs)-positive diffuse redness as diffuse redness score 1, and severe diffuse redness or non-RAC diffuse redness as diffuse redness score. Endoscopic findings of map-like redness were defined as an erythematous lesion with a shallow depression and boundaries distinct from the background mucosa, and the size and location of the map-like redness were evaluated. Hiatal hernia was endoscopically diagnosed when the esophagogastric junction was dislocated toward the esophageal site by more than 2 cm^24^.

Two expert endoscopists (SM and IE), who are certified as endoscopists by the Japan Endoscopic Society, independently evaluated the endoscopic findings using medical records, while being blinded regarding both the diagnosis and clinical information of the patients. When the severity of the endoscopic findings assigned by the 2 endoscopists differed, the mean value was used.

### *H. pylori* infection diagnosis and eradication therapy

*H. pylori* infection was diagnosed in all patients using the ^13^C-urea breath test (UBIT100 mg tablets, Otsuka Pharmaceutical Co., Ltd., using a cutoff of 2.5‰), the rapid urease test (Helicocheck®; Institute of Immunology, Co., Ltd.,), and an anti-*H. pylori* IgG test (antibody determination kit, E-Plate Eiken *H*. *pylori* antibody, using a cut-off of 10 U/mL). Patients were diagnosed as *H. pylori* infection-positive if they scored positive in at least 1 of the 3 tests.

### Statistical analysis

Values for age and scores of the Kyoto Classification and hiatal hernia are shown as the mean ± standard deviation. Statistical significance of the differences in categorical data among the 3 groups was determined by the χ^2^ test. The Wilcoxon signed-rank test was used to compare Kyoto classification scores from the first endoscopy, and those 5 years, 10 years, 15 years, and 20 years subsequently. A *p*-value of less than 0.05 was considered to indicate a statistically significant difference between groups, and all *p*-values were 2-sided. Calculations were conducted using STATA version 17 software (StataCorp, College Station, TX).

## Results

### Characteristics of the patients

Of the 172 patients who underwent endoscopic follow-up for more than 10 years, 3 patients who underwent surgery for gastric cancer and 2 patients whose result of eradication was unknown were excluded, and 167 patients were finally included in this study (Table 1).

**Table 1:** Baseline characteristics of the patients.

	Total N = 167	Eradication group N = 143	Current infection group N = 4	Never infection group N = 20	*P*-value
Age (years, mean ± SD)	74.7 ± 8.5	75.4 ± 7.7	80.5 ± 6.5	69.1 ± 11.2	0.016
Sex, male, n (%)	107 (64.1%)	94 (65.7%)	3 (75.0%)	10 (50%)	0.350
Smoking history, n (%)					
Nonsmoker	114 (68.3%)	97 (67.8%)	2 (50.0%)	15 (75.0%)	0.442
Current/ex-smoker	53 (31.7%)	46 (32.2%)	2 (50.0%)	5 (25.0%)	
Alcohol consumption (n [%])					
Never/rare drinker	89 (53.3%)	77 (53.8%)	2 (50.0%)	10 (50.0%)	0.128
Current/ex-drinker	78 (46.7%)	66 (46.2%)	2 (50.0%)	10 (50.0%)	
Family history, n (%)					
Any cancer	105 (62.9%)	95 (66.4%)	1 (25.0%)	9 (45.0%)	0.196
Gastric cancer	37 (22.2%)	35 (24.5%)	0 (0.0%)	2 (10.0%)	0.285
Previous history of cancer, n (%)					
Any cancer	66 (39.5%)	62 (43.4%)	3 (75.0%)	1 (5.0%)	0.002
Gastric cancer	44 (26.4%)	41 (28.7%)	3 (75.0%)	0 (0.0%)	0.002
Observation period (years, mean ± SD)	15.7 ± 3.1	15.8 ± 3.1	16.5 ± 3.3	14.7 ± 3.4	0.337

S.D., standard deviation.

Eradication group, patients who were previously infected with but were successfully eradicated of *H. pylori*; Current infection group, patients who had not undergone eradication during the follow-up period; Never infection group, patients with no endoscopic, pathological, or serological signs of *H. pylori* infection.

The mean age of all patients was 74.7 ± 8.5 years, and 64.1% were men. There were 143 patients in the Eradication group (patients in whom successful eradication was performed during the period, or eradication was completed at the beginning), 4 patients in the Current infection group (patients who currently had *H. pylori* infection), and 20 patients in the Never infection group (patients who had never been infected with *H. pylori*). Four patients in the current infection group were not given eradication treatment because they had no consent to receive eradication therapy. A significant difference was observed between each group in age and past history of cancer, but no significant difference was observed in sex, smoking, alcohol consumption, and family history of cancer (Table 1). The average period of endoscopic observation was 15.7 ± 3.1 years, with no significant difference between the groups.

### Change of *H. pylori* status and severity of gastritis during the follow-up period

**Figure 1:**
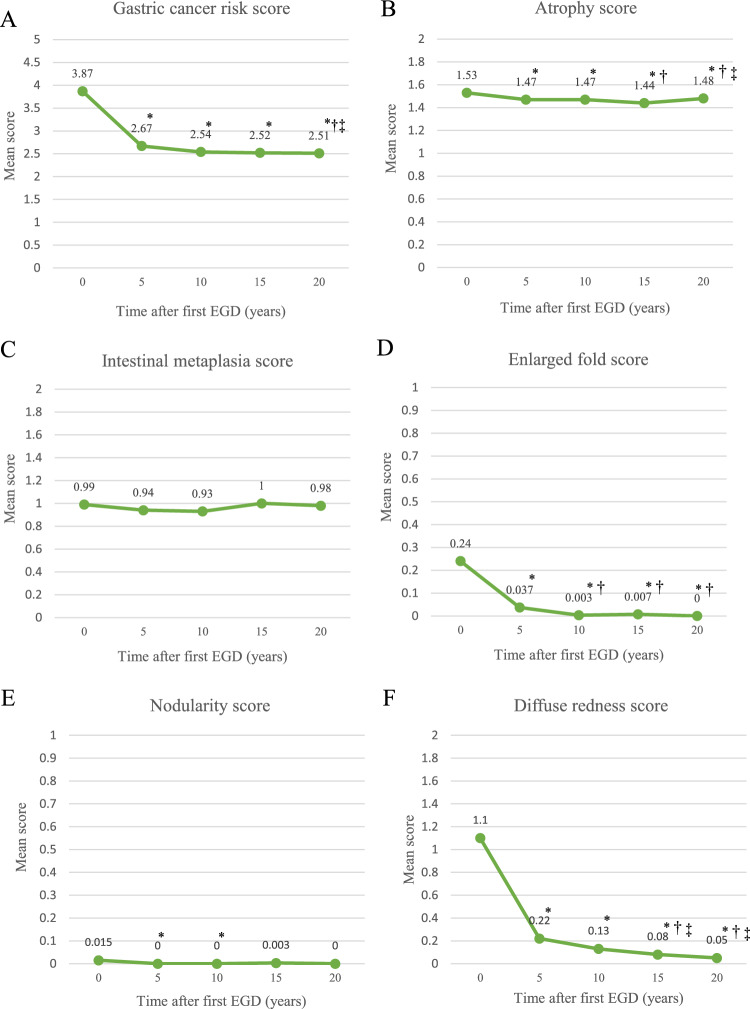
Long-term changes in gastritis scores based on the Kyoto Classification of Gastritis in all patients. (**A**) Mean total score of gastric cancer risk. (**B**) Mean atrophy score. (**C**) Mean intestinal metaplasia score. (**D**) Mean fold enlargement score. (**E**) Mean nodularity score. (**F**) Mean diffuse redness score. Significant improvements in total gastric cancer risk score and scores for atrophy, fold enlargement, and diffuse redness were observed in all patients up to 20 years after *H. pylori* eradication. No significant score change was observed for intestinal metaplasia. *: *p* < 0.05 versus first EGD, †: versus EGD 5 years subsequently, and ‡: versus EGD 10 years subsequently.

Of the 107 *H. pylori*-positive patients at baseline, eradication was completed in most patients (81 patients) within 5 years after EGD (Supplementary Table 1). Compared with the total gastric cancer risk scores of the Kyoto Classification of Gastritis at the first EGD, significant improvements in mean total gastric cancer risk score (3.87 ± 2.08 to 2.51 ± 1.29, *p* < 0.001), and mean scores for atrophy (1.53 ± 0.68 to 1.48 ± 0.65, *p* = 0.008), fold enlargement (0.24 ± 0.35 to 0.00 ± 0.00, *p* < 0.001), and diffuse redness (1.10 ± 0.84 to 0.05 ± 0.24, *p* < 0.001) were observed in all patients 20 years subsequently, and their scores improved over time (Fig. 1, Supplementary Table 2). No significant score change was observed for intestinal metaplasia. Improvement of most risk scores including gastric cancer risk score, atrophy score, enlarged fold score and diffuse redness score occurred within 5 years after initial endoscopic observation and those scores are stable thereafter (Fig. 1).

### Changes in Kyoto Classification scores among the 3 different *H. pylori* status groups

**Figure 2:**
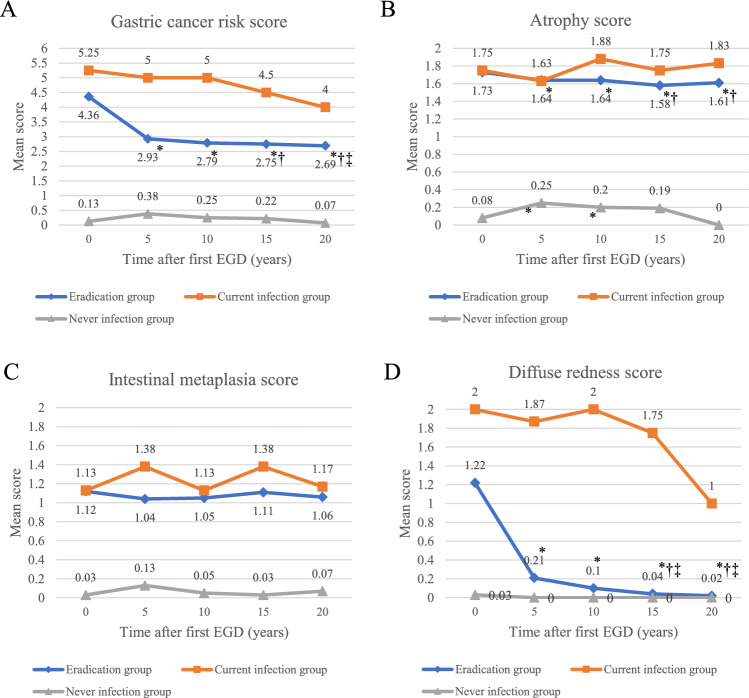
Prolonged changes in gastritis scores based on the Kyoto Classification of Gastritis among the Eradication group, Current infection group, and Never infection group. (**A**) Mean total score of gastric cancer risk. (**B**) Mean atrophy score. (**C**) Mean intestinal metaplasia score. (**D**) Mean diffuse e redness score. Significant improvements in total gastric cancer risk, atrophy, and diffuse redness scores compared with the first EGD were observed in the Eradication group. There was no change in scores in the Never infection group. In the Current infection group, progression of the scores for atrophy and intestinal metaplasia were not observed. *: *p* < 0.05 versus first EGD, †: versus EGD 5 years subsequently, and ‡: versus EGD 10 years subsequently.

In the Eradication group, there was a significant improvement in mean total gastric cancer risk (4.36 ± 1.66 to 2.69 ± 1.07, *p* < 0.001), atrophy (1.73 ± 0.44 to 1.61 ± 0.49, *p* = 0.004), and diffuse redness scores (1.22 ± 0.79 to 0.02 ± 0.13, *p* < 0.001) compared with the first EGD, and improvements in scores with time were also observed (Fig. 2, Supplementary Table 3). There was no change in mean score with time for each item in the Never infection group. In the Current infection group, progression of the mean scores for atrophy and intestinal metaplasia were not observed. Improvement of mean gastric cancer risk score, atrophy score, and mean diffuse redness score occurred within 5 years in patients eradicated *H. pylori* infection and those scores are stable thereafter (Fig. 2).

### Changes in frequency of map-like redness

**Figure 3:**
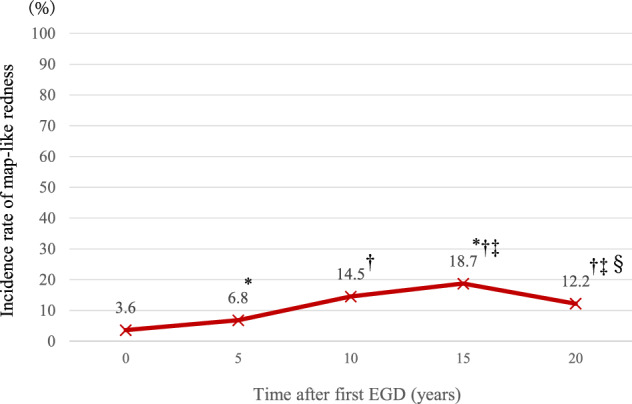
Incidence rate of map-like redness during the long-term observation period. The frequency of map-like redness in all patients increased significantly over time from the first EGD up to 15 years subsequently. The frequency of map-like redness 20 years after eradication was significantly decreased compared with that 15 years after eradication. *: *p* < 0.05 versus first EGD, †: versus 5 years subsequently, ‡: versus 10 years subsequently, and §: versus 15 years subsequently

The frequency of map-like redness in all patients increased significantly over time from the first EGD up to 15 years subsequently (3.6% to 18.7%, *p* = 0.03), according to the increase in prevalence of patients in the Eradication group (Fig. 3, Supplementary Table 4). The frequency of map-like redness 20 years after eradication was significantly decreased compared with those 15 years after eradication (18.7% to 12.2%, *p* < 0.001) (Fig. 3).

### Changes in hiatal hernia score

All patients demonstrated a significant increase in hiatal hernia score over time (0.55 ± 0.71 to 1.22 ± 0.88, *p* < 0.001) (Supplementary Fig. 1). The Eradication group, Current infection group, and Never infection group all showed a similar increase in scores over time.

### Kyoto classification score and rate of map-like redness in the cancer group and noncancer group

There were 45 patients who developed gastric cancer during the observation period. The group that developed gastric cancer was designated as the Cancer group, and the group that did not develop gastric cancer as the Noncancer group.

**Figure 4:**
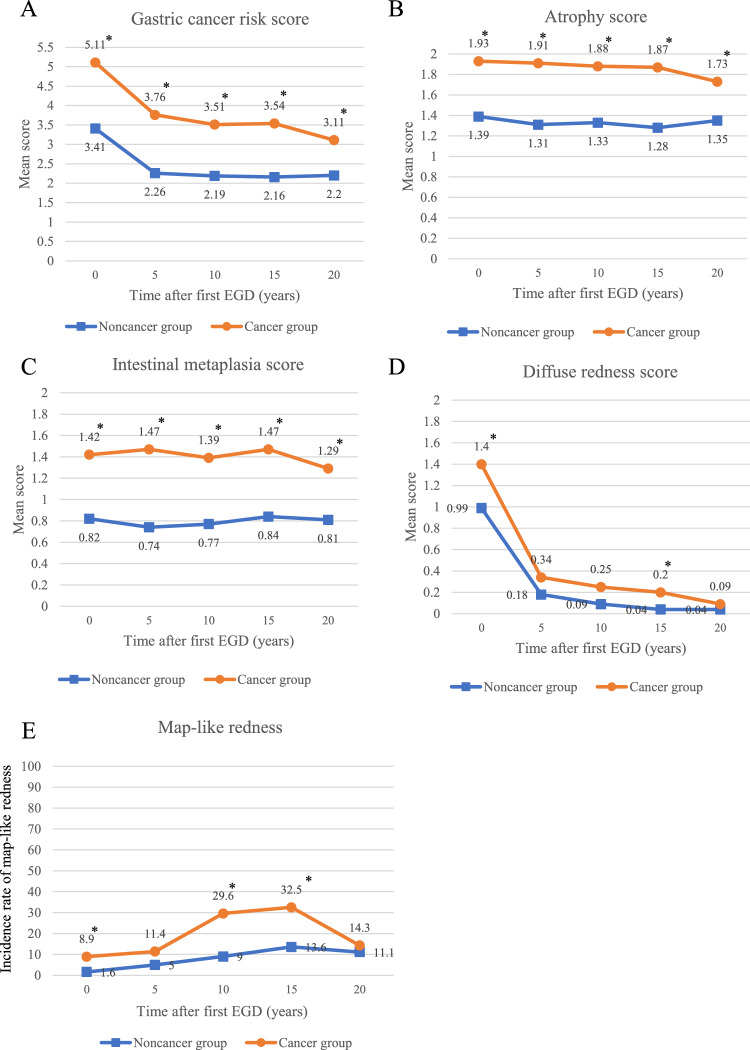
Prolonged changes in gastritis scores based on the Kyoto Classification of Gastritis between the Cancer group and the Noncancer group. (**A**) Mean total score of gastric cancer risk. (**B**) Mean atrophy score. (**C**) Mean intestinal metaplasia score. (**D**) Mean diffuse redness score. (**E**) Incidence rate of map-like redness The Cancer group had significantly higher total risk scores, and scores for atrophy and intestinal metaplasia at all time points than the Noncancer group. Regarding map-like redness, the frequency increased with time from the first EGD to 15 years subsequently in both the Cancer group and the Noncancer group. The Cancer group had a higher frequency of map-like redness at all time points than the Noncancer group. *: *p* < 0.05 versus Noncancer group.

In the association with severity of gastric atrophy and location of gastric cancer, most of patient with gastric cancer had moderate (Kimura-Takemoto classification: C3 to O1) to severe atrophy (Kimura-Takemoto classification: O2 to O3) (Table 2). Only one patient of gastric cancer development was observed from patients with mild atrophy. Compared with the Noncancer group, the Cancer group had significantly higher total risk scores, and more severe atrophy and intestinal metaplasia at all time points (Fig. 4). The mean total risk score (5.11 ± 1.18 to 3.11 ± 1.03, *p* < 0.001), atrophy (1.93 ± 0.25 to 1.73 ± 0.46, *p* = 0.049), and diffuse redness (1.40 ± 0.77 to 0.09 ± 0.27, *p* < 0.001) scores significantly improved over time in the Cancer group (Fig. 4, Supplementary Table 5). In the Noncancer group, mean total gastric cancer risk score (3.41 ± 2.16 to 2.20 ± 1.30, *p* < 0.001) and diffuse redness score (0.99 ± 0.84 to 0.04 ± 0.21, *p* < 0.001) significantly improved over time. Regarding intestinal metaplasia, no change was observed in either group.

**Table 2:** Association with gastric cancer location and severity of atrophy.

Location of gastric cancer	Total number N = 45	Kimura-Takemoto C0	Kimura-Takemoto C1-2	Kimura-Takemoto C3-O1	Kimura-Takemoto O2-3
Antrum	11	0	1	5	5
Angle	11	0	0	5	6
Lower body	8	0	0	6	2
Middle body	9	0	0	8	1
Upper body	4	0	0	3	1
Cardia	1	0	0	0	1
Fornix	1	0	0	0	1

Regarding map-like redness, the frequency increased over time from the first EGD to 15 years subsequently in both the Cancer (8.9% to 32.5%, *p* = 0.59) and the Noncancer groups (1.6% to 13.6%, *p* < 0.001), and decreased after 20 years (Cancer group: 32.5% to 14.3%, *p* = 0.002; Noncancer group: 13.6% to 11.1%, *p* < 0.001). In addition, the Cancer group had a higher frequency of map-like redness at all time points than the Noncancer group.

The time courses of mean atrophy, intestinal metaplasia, enlarged fold, disuse redness and map-like redness scores between patients with or without a history of gastric cancer (Supple Fig. 2) and those who developed gastric cancer or not (Fig. 4) were a similar.

### Changes among different severity of atrophy and intestinal metaplasia according time-course

**Figure 5:**
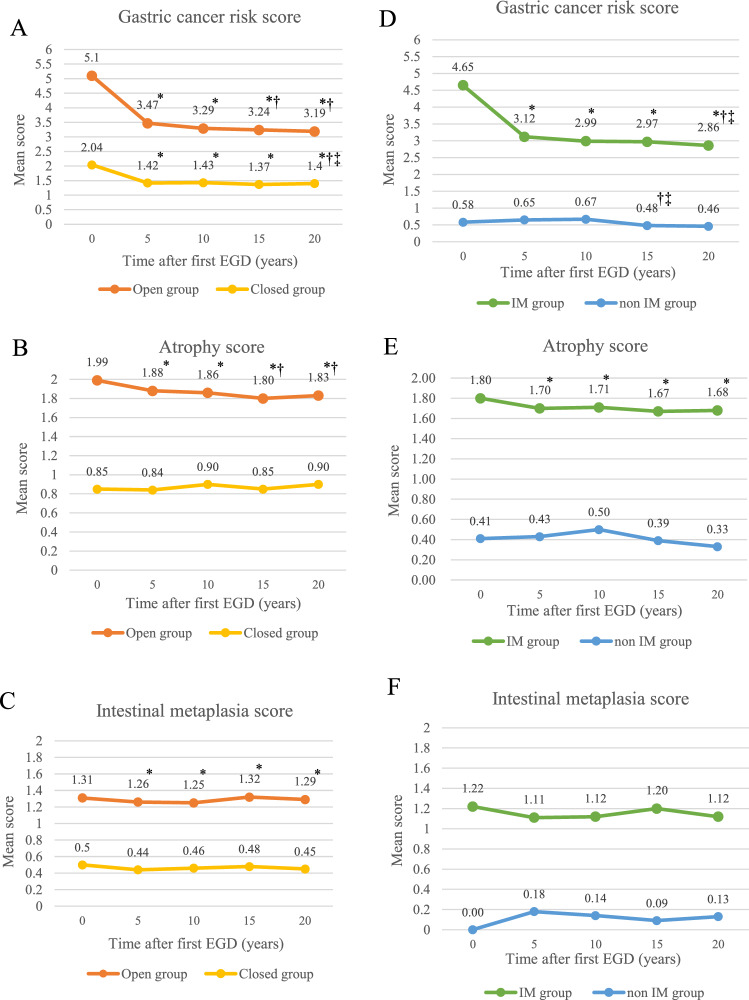
Changes among different severity of atrophy and intestinal metaplasia according time-course. Mean gastric cancer risk score, mean atrophy and mean intestinal metaplasia scores in both patients with open type atrophy according to the Kimura-Takemoto classification (**A**, **B**, **C**) and patients with intestinal metaplasia (**D**, **E**, **F**) decreased with the passage of time. *: *p* < 0.05 versus first EGD, †: versus EGD 5 years subsequently, and ‡: versus EGD 10 years subsequently.

Mean gastric cancer risk score and atrophy scores in patients with open type atrophy according to the Kimura-Takemoto classification and patients with intestinal metaplasia decreased with the passage of time (Fig. 5, Supplementary Table 6). In patients with closed type, only mean gastric cancer risk score improved significantly. For patients without intestinal metaplasia, no significant changes were observed in any score. No significant changes were observed in the intestinal metaplasia score in any group.

### Predictive factors for gastric cancer

In univariate analysis, predictive factors were sex, past history of cancer, past history of gastric cancer, current *H. pylori* infection, Kimura-Takemoto classification open-type, Kyoto classification of gastritis mean atrophy, intestinal metaplasia, fold enlargement, diffuse redness, total score, and map-like redness (Table 3).

**Table 3:** Univariate analysis and multivariate analysis for gastric cancer development.

Factors	Univariate analysis		Multivariate analysis	
	OR (95% CI)	*P* value	OR (95% CI)	*P* value
Age	1.03 (0.99–1.07)	0.171		
Sex (vs. female)	3.43 (1.48–7.99)	< 0.001	2.10 (0.45–9.76)	0.342
Smoking	1.14 (0.78–1.69)	0.493		
Drinking	0.93 (0.73–1.19)	0.558		
Family history of cancer	1.06 (0.53–2.11)	0.877		
Number of cancers in family history	0.99 (0.72–1.37)	0.960		
Family history of gastric cancer	1.10 (0.47–2.53)	0.824		
Number of gastric cancers in family history	1.24 (0.63–2.44)	0.543		
Past history of cancer	29.5 (10.59–82.39)	< 0.001		
Past history of gastric cancer	236 (60.40–922.08)	< 0.001	157.6 (35.61–697.6)	< 0.001
Current *H. pylori* infection	2.58 (1.06–6.33)	0.037		
Eradication therapy	3.08 (0.87–10.84)	0.080		
Duration after eradication (years)	0.96 (0.88–1.05)	0.387		
At endoscopic findings in first endoscopy				
Kimura-Takemoto classification (vs. close type)	3.07 (1.86–5.04)	< 0.001		
Kyoto classification of gastritis				
Atrophy	12.8 (3.32–49.56)	< 0.001	1.83 (0.31–10.9)	0.508
Intestinal metaplasia	4.76 (2.50–9.08)	< 0.001	1.09 (0.26–4.57)	0.905
Fold enlargement	3.01 (1.18–7.66)	0.021		
Nodularity	0.45 (0.01–37.99)	0.724		
Diffuse redness	1.88 (1.20–2.94)	< 0.001		
Total score	1.70 (1.33–2.17)	< 0.001		
Map-like redness	5.85 (1.03–33.15)	0.046	1.74 (0.05–60.7)	0.759

Of possible predictive factors showing a p value < 0.05 in univariate analysis, although the number of events (gastric cancer) is 45 which is relatively small, we included five factors (male sex, past history of gastric cancer, Kyoto classification of gastritis atrophy, intestinal metaplasia and map-like redness) which were generally known to be major risk factors for gastric cancer. On multivariate analysis, risk factor was past history of gastric cancer [OR: 157.6 (35.61–697.6)] (Table 3).

## Discussion

In this study, we demonstrated that among the risk factors stated in the Kyoto Classification of Gastritis, atrophy significantly improved in patients eradicated of *H. pylori*, over the long-term after eradication. On the other hand, although intestinal metaplasia is considered to be a major risk factor for gastric cancer, it did not change throughout this long time period. As an important point of this study, we found that patients who developed gastric cancer during the observation period had a high total risk score according to the Kyoto Classification of Gastritis, as well as severe atrophy and severe intestinal metaplasia compared with those who did not develop gastric cancer. In both the Cancer group and the Noncancer group, changes in total risk score, atrophy, and intestinal metaplasia were similar. Therefore, we believe that the risk of gastric cancer after eradication can be assessed by endoscopic factors, such as atrophy, intestinal metaplasia, and map-like redness, and that the total risk score according to the Kyoto Classification of Gastritis is useful for identifying patients at high-risk of developing gastric cancer.

The MAPS II guidelines, which explains how to manage epithelial precancerous conditions and lesions in the stomach, state that patients with atrophic gastritis or intestinal metaplasia are at a high risk for gastric cancer (Agree: 100%, and Evidence level: High)^13^. In fact, of the patients followed for up to 14.1 years, the incidence rates of gastric cancer differed among patients with different severities of atrophy; the rate of gastric cancer in patients eradicated of *H. pylori* infection was 0.04%/year in patients with mild atrophy, 0.28%/year in those with moderate atrophy, and 0.62%/year in those with severe atrophy^25^. A meta-analysis using prospective case–control studies showed a significant association between the OLGA stages, pathological evaluation system of atrophy, and gastric cancer risk^26^. OLGA stages III/IV were associated with an increased risk of gastric cancer in terms of odds ratio (2.41; 95% CI 2.02–2.88; *p* < 0.00001) compared with OLGA stages I/II^26^. Therefore, endoscopic and pathological assessment of atrophy is considered to be important for the stratification of gastric cancer risk.

Improvement of atrophy and intestinal metaplasia is generally considered to reduce gastric cancer risk. A previous meta-analysis investigated the association between *H. pylori* eradication and the histological improvement of atrophy, as a long-term effect of *H. pylori* eradication on gastric histology^27–29^. The pooled weighted mean difference in the gastric antrum and corpus before and after eradication was 0.25 (95% CI 0.15–0.35) and 0.14 (95% CI 0.04–0.24), with a significant overall effect (both *p* < 0.05)^28^. Because East-Asian countries have high incidences of gastric cancer, it is possible that people in East-Asia who are infected with *H. pylori* have a higher rate of advanced atrophy, and hence improvements of atrophy and intestinal metaplasia should be considered to be different between East-Asian and Western populations. In a long-term observation study (up to 17 years after *H. pylori* eradication) in Japanese patients, Kodama, et al.^30^ reported that relative to the baseline before eradication, pathological atrophy scores according to the updated Sydney system had improved significantly 1 year after eradication in the antrum (1.50 ± 0.75 vs. 1.21 ± 1.25, *p* < 0.01) and corpus (0.59 ± 0.75 vs. 0.18 ± 0.52, *p* < 0.05). The improvement of these scores continued to decrease for the next 10 years and remained stable thereafter. In the present study, we demonstrated a significant improvement in endoscopic, but not histopathological gastric cancer risk, as well as improvements in atrophy and diffuse redness compared with the first EGD in patients after eradication. In addition, progression or improvement of intestinal metaplasia score was not observed during the mean observation period of 15.7 years. This result suggested that the improvement of atrophy after eradication reduces the risk of gastric cancer development, and the risk decreases over time. It is important to know whether it is better to evaluate atrophy and intestinal metaplasia by pathological evaluation or endoscopic evaluation. Toyoshima et al. reported that endoscopic Kyoto Classification Scores determined by endoscopic analyses and Updated Sydney System Scores determined by pathological analyses were consistent in *H. pylori*-positive Japanese individuals, regarding the level of atrophy and intestinal metaplasia^31^. Histological evaluation by biopsies cannot be performed in all patients, owing to its cost and risk of post-biopsy bleeding. Previously published meta-analyses evaluated atrophy and intestinal metaplasia based on pathology, but in our study, we evaluated endoscopic findings, which are actually useful in clinical practice for all patients. In addition, although the average follow-up period was 15.7 years in this study, we were able to evaluate long-term progression in many patients; i.e., 150 patients (89.8%) were evaluated for 15 years or more, and 82 patients (49.1%) were evaluated for 20 years. We believe that the results of our study show the usefulness of endoscopic long-term follow-up for the risk assessment of gastric cancer after eradication, in addition to the pathological evaluation system.

In general, diffuse redness, enlarged fold, and nodularity, which are indicative of current *H. pylori* infection, in the gastric cancer risk score are rapidly improved after eradication therapy, while atrophy gradually improves throughout a long time after eradication^18,30,32^. Gastric cancer after eradication is often detected within a few years after eradication, and the incidence of cancer after eradication is known to gradually decrease over the course of the post-eradication period^33^. In fact, this recent trial showed that improvement of gastric cancer risk score and diffuse redness score occurred within 5 years in patients eradicated *H. pylori* infection and those scores are stable thereafter. Given that severity of gastritis will progress in the situation not received eradication therapy in patients with current infection, the benefits of eradication therapy for gastric chemoprevention may occur several years after eradication. Because the International guidelines for *H. pylori* infection in the US, Europe and Asian countries strongly recommend eradication of *H. pylori* to prevent the development of gastric cancer^3,4,34,35^, eradication treatment and long-term surveillance are considered important in clinical practice for gastric cancer.

Map-like redness has attracted attention as a risk factor and predictor of gastric cancer after eradication^36^. Majima et al.^36^ reported that on multivariate analysis, map-like redness was an independent risk factor (observed by white-light imaging [WLI]: odds ratio 2.05; 95% CI 1.09–3.87; *p* = 0.03; observed by linked-color imaging: OR 3.62; 95% CI 1.88–6.97; *p* < 0.001), whereas the regular arrangement of collecting venules was an independent negative risk factor for the new detection of metachronous gastric cancer after eradication. Moribata et al.^37^ reported that patients who developed map-like redness after eradication were significantly more likely to develop metachronous gastric cancer (64%, 14/22) than those without map-like redness (25%, 25/100). Many clinical studies have demonstrated that map-like redness is a characteristic endoscopic risk factor for gastric cancer in patients who received eradication therapy, but studies regarding the timing and incidence of map-like redness are limited. In the present study, incidence rates of map-like redness in patients after eradication were 7.4% at 5 years, 17.3% at 10 years, and 20.2% at 15 years after eradication. The development of map-like redness may increase with the passage of time after eradication, suggesting that gastric cancer risk changes with the passage of time after eradication. In addition, it is thought that how long it takes for map-like redness to develop depends on the individual patient, so it is necessary to investigate long-term changes in a larger number of patients than analyzed in this study.

Pathologically severe intestinal metaplasia group (e.g., OLGIM stages 3 and 4) had the high risk for gastric cancer development^13,38^. In this study, because we evaluate severity of intestinal metaplasia by endoscopy, we had no data concerning pathological severity of intestinal metaplasia. Of those, we think that the important issue is whether the endoscopic evaluation of gastritis is consistent with the histopathologic evaluation^21,31,39,40^. In one study, endoscopic findings based on the Kyoto classification of gastritis (*H. pylori*-positive, 303 patients; *H. pylori*-negative, 258 patients) were shown to have 98.7% sensitivity and 98.4% specificity for histological gastritis^40^. Toyoshima et al.^31^ reported that endoscopic atrophy in the corpus was associated with high scores for pathological atrophy using the updated Sydney system (*p* = 0.049) and that endoscopic intestinal metaplasia in both the corpus and antrum was associated with high scores of pathological intestinal metaplasia (all *p* < 0.001). These observations suggest that atrophy and intestinal metaplasia are consistent between endoscopic and pathological diagnoses. However, the lack of histopathological evaluation can be considered as a limitation of our study and is considered to be an issue for the future study.

There are some limitations to this study. First, this study was a retrospective observational study at a single institution. Second, because recent developments in endoscopic instrumentation and image-enhancement techniques, known together as image-enhanced endoscopy (IEE), including narrow-band imaging with or without magnification, have improved the detection rate of gastric cancer, atrophy, and intestinal metaplasia, the accuracy of endoscopic equipment is changing every year^41,42^. Examinations performed in earlier years were evaluated by only WLI, whereas those performed recently also use IEE, so it is possible that more regions of intestinal metaplasia and map-like redness are now detected. Third, we have no data about the pathological evaluation of gastric condition. Fourth, the influence of gastrin level associated with atrophic changes in the development of gastric cancer cannot be ignored. In a view of gastric cancer development, it would be appropriate to evaluate histopathological changes and changes of serum gastric level along with long-term changes in endoscopic findings, but we had no data of histopathology and gastric level due to a retrospective study. Fifth, two-thirds of patients developed gastric cancer or other cancers, because it is relatively high compared with other studies, this study may have selection bias. This study however is an observational study with up to 20 years of follow-up and patients was elderly patients with high-risk for cancer.

In this study, we demonstrated that *H. pylori* eradication therapy significantly changed the gastric condition of patients, including atrophy, which is a major risk factor for gastric cancer. This observation suggests that eradication therapy for H. pylori infection is one of the key elements in gastric cancer prevention during long-term periods, more than 10 years. Therefore, because time-dependent changes in severity and status of risk factors for gastric cancer occur after eradication, we believe that it is crucial to pay careful attention to such changes during surveillance endoscopy. Not only the severity of gastritis (e.g., atrophy and intestinal metaplasia) on endoscopic surveillance, but also the severity of gastritis before eradication (e.g., score of the Kyoto Classification of Gastritis), and the presence of map-like redness after eradication should be taken into account in assessing the risk of gastric cancer occurrence.

### Supplementary Information

Supplementary Tables.

Supplementary Figures.

## Data Availability

The data based on the results of the current study were obtained, are accessible from the corresponding authors upon reasonable request.
