# Supplementary material for: Long-term endoscopic gastric mucosal changes up to 20 years after Helicobacter pylori eradication therapy
Source: Sci Rep. 2024 Jun 6;14:13003. doi: 10.1038/s41598-024-63928-6 (PMC11156848; doi:10.1038/s41598-024-63928-6)
Supplement: Supplementary file 1 — Supplementary Tables. [file 41598_2024_63928_MOESM1_ESM.docx]

**Supplementary table 1. *Helicobacter pylori* status of patients at each time point**

| Group | First EGD  N = 167 | 5 years subsequently  N = 163 | 10 years subsequently  N = 166 | 15 years subsequently  N = 150 | 20 years subsequently  N = 82 |
| --- | --- | --- | --- | --- | --- |
| Past infection | 40 | 121 | 133 | 124 | 71 |
| Current infection | 107 | 22 | 13 | 10 | 4 |
| Never infection | 20 | 20 | 20 | 16 | 7 |

**Supplementary table 2. Long-term changes in gastritis scores based on the Kyoto Classification of Gastritis in all patients (Figure1 data)**

| Score | | | Mean | S.D. |
| --- | --- | --- | --- | --- |
| Gastric cancer risk score | | |  |  |
|  | Time after first EGD | 0 | 3.87 | 2.08 |
|  |  | 5 | 2.67 | 1.51 |
|  |  | 10 | 2.54 | 1.34 |
|  |  | 15 | 2.52 | 1.34 |
|  |  | 20 | 2.51 | 1.29 |
| Atrophy score | | |  |  |
|  | Time after first EGD | 0 | 1.53 | 0.68 |
|  |  | 5 | 1.47 | 0.66 |
|  |  | 10 | 1.47 | 0.64 |
|  |  | 15 | 1.44 | 0.64 |
|  |  | 20 | 1.48 | 0.65 |
| Intestinal metaplasia score | | |  |  |
|  | Time after first EGD | 0 | 0.99 | 0.67 |
|  |  | 5 | 0.94 | 0.69 |
|  |  | 10 | 0.93 | 0.67 |
|  |  | 15 | 1.00 | 0.72 |
|  |  | 20 | 0.98 | 0.72 |
| Enlarged fold score | | |  |  |
|  | Time after first EGD | 0 | 0.24 | 0.35 |
|  |  | 5 | 0.037 | 0.153 |
|  |  | 10 | 0.003 | 0.039 |
|  |  | 15 | 0.007 | 0.058 |
|  |  | 20 | 0.00 | 0.00 |
| Nodularity score | | |  |  |
|  | Time after first EGD | 0 | 0.015 | 0.085 |
|  |  | 5 | 0.00 | 0.00 |
|  |  | 10 | 0.00 | 0.00 |
|  |  | 15 | 0.003 | 0.041 |
|  |  | 20 | 0.00 | 0.00 |
| Diffuse redness score | | |  |  |
|  | Time after first EGD | 0 | 1.10 | 0.84 |
|  |  | 5 | 0.22 | 0.54 |
|  |  | 10 | 0.13 | 0.39 |
|  |  | 15 | 0.08 | 0.36 |
|  |  | 20 | 0.05 | 0.24 |

S.D.: standard deviation, EGD: Esophagogastroduodenoscopy

**Supplementary table 3. Prolonged changes in gastritis scores based on the Kyoto Classification of Gastritis among the Eradication group, Current infection group, and Never infection group**

|  | | | Eradication group (n=143) | | Current Infection group (n=4) | | Never infection group (n=20) | |
| --- | --- | --- | --- | --- | --- | --- | --- | --- |
|  | | | Mean | S.D | Mean | S.D | Mean | S.D |
| Gastric cancer risk score | | |  |  |  |  |  |  |
|  | Time after first EGD | 0 | 4.36 | 1.66 | 5.25 | 0.65 | 0.13 | 0.36 |
|  |  | 5 | 2.93 | 1.26 | 5.00 | 0.58 | 0.38 | 0.81 |
|  |  | 10 | 2.79 | 1.05 | 5.00 | 0.41 | 0.25 | 0.41 |
|  |  | 15 | 2.75 | 1.11 | 4.50 | 0.71 | 0.22 | 0.41 |
|  |  | 20 | 2.69 | 1.07 | 4.00 | 1.00 | 0.07 | 0.19 |
| Atrophy score | | |  |  |  |  |  |  |
|  | Time after first EGD | 0 | 1.73 | 0.44 | 1.75 | 0.50 | 0.08 | 0.18 |
|  |  | 5 | 1.64 | 0.48 | 1.63 | 0.48 | 0.25 | 0.41 |
|  |  | 10 | 1.64 | 0.46 | 1.88 | 0.25 | 0.20 | 0.30 |
|  |  | 15 | 1.58 | 0.49 | 1.75 | 0.50 | 0.19 | 0.31 |
|  |  | 20 | 1.61 | 0.49 | 1.83 | 0.29 | 0.00 | 0.00 |
| Intestinal metaplasia score | | |  |  |  |  |  |  |
|  | Time after first EGD | 0 | 1.12 | 0.61 | 1.13 | 0.25 | 0.03 | 0.11 |
|  |  | 5 | 1.04 | 0.64 | 1.38 | 0.25 | 0.13 | 0.46 |
|  |  | 10 | 1.05 | 0.63 | 1.13 | 0.25 | 0.05 | 0.15 |
|  |  | 15 | 1.11 | 0.68 | 1.38 | 0.48 | 0.03 | 0.13 |
|  |  | 20 | 1.06 | 0.69 | 1.17 | 0.76 | 0.07 | 0.19 |
| Diffuse redness score | | |  |  |  |  |  |  |
|  | Time after first EGD | 0 | 1.22 | 0.79 | 2.00 | 0.00 | 0.03 | 0.11 |
|  |  | 5 | 0.21 | 0.50 | 1.87 | 0.25 | 0.00 | 0.00 |
|  |  | 10 | 0.10 | 0.28 | 2.00 | 0.00 | 0.00 | 0.00 |
|  |  | 15 | 0.04 | 0.24 | 1.75 | 0.50 | 0.00 | 0.00 |
|  |  | 20 | 0.02 | 0.13 | 1.00 | 0.50 | 0.00 | 0.00 |

S.D.: standard deviation, EGD: Esophagogastroduodenoscopy

**Supplementary table 4. Incidence rate of map-like redness during the long-term observation period**

|  | | | map-like redness | | | | |
| --- | --- | --- | --- | --- | --- | --- | --- |
|  | | | n | Number of positives | Incidence rate | 95% CI | |
| Time after first EGD | | 0 | 167 | 6 | 0.036 | 0.033 | 0.041 |
|  |  | 5 | 163 | 11 | 0.068 | 0.064 | 0.073 |
|  |  | 10 | 166 | 24 | 0.145 | 0.137 | 0.153 |
|  |  | 15 | 150 | 28 | 0.187 | 0.177 | 0.198 |
|  |  | 20 | 82 | 10 | 0.122 | 0.112 | 0.135 |

95% CI: 95% Confidence Interval

**Supplementary table 5. Prolonged changes in gastritis scores based on the Kyoto Classification of Gastritis between the Cancer group and the Noncancer group**

|  | | | Cancer group (n=143) | | Non cancer group (n=4) | |
| --- | --- | --- | --- | --- | --- | --- |
| Score | | | Mean | S. D | Mean | S. D |
| Gastric cancer risk score | | |  |  |  |  |
|  | Time after first EGD | 0 | 5.11 | 1.18 | 3.41 | 2.16 |
|  |  | 5 | 3.76 | 0.92 | 2.26 | 1.49 |
|  |  | 10 | 3.51 | 0.95 | 2.19 | 1.29 |
|  |  | 15 | 3.54 | 0.86 | 2.16 | 1.31 |
|  |  | 20 | 3.11 | 1.03 | 2.20 | 1.30 |
| Atrophy score | | |  |  |  |  |
|  | Time after first EGD | 0 | 1.93 | 0.25 | 1.39 | 0.73 |
|  |  | 5 | 1.91 | 0.22 | 1.31 | 0.69 |
|  |  | 10 | 1.88 | 0.31 | 1.33 | 0.67 |
|  |  | 15 | 1.87 | 0.27 | 1.28 | 0.66 |
|  |  | 20 | 1.73 | 0.46 | 1.35 | 0.70 |
| Intestinal metaplasia score | | |  |  |  |  |
|  | Time after first EGD | 0 | 1.42 | 0.53 | 0.82 | 0.65 |
|  |  | 5 | 1.47 | 0.53 | 0.74 | 0.63 |
|  |  | 10 | 1.39 | 0.53 | 0.77 | 0.64 |
|  |  | 15 | 1.47 | 0.54 | 0.84 | 0.71 |
|  |  | 20 | 1.29 | 0.69 | 0.81 | 0.68 |
| Diffuse redness score | | |  |  |  |  |
|  | Time after first EGD | 0 | 1.40 | 0.77 | 0.99 | 0.84 |
|  |  | 5 | 0.34 | 0.66 | 0.18 | 0.48 |
|  |  | 10 | 0.25 | 0.58 | 0.09 | 0.29 |
|  |  | 15 | 0.20 | 0.53 | 0.04 | 0.27 |
|  |  | 20 | 0.09 | 0.27 | 0.04 | 0.21 |

|  | | | Cancer group (n=143) | | | | | Non cancer group (n=4) | | | | |
| --- | --- | --- | --- | --- | --- | --- | --- | --- | --- | --- | --- | --- |
|  | | | n | positive | rate | 95% CI | | n | positives | rate | 95% CI | |
| map-like redness | | |  |  |  |  |  |  |  |  |  |  |
|  | Time after first EGD | 0 | 45 | 4 | 0.089 | 0.077 | 0108 | 122 | 2 | 0.016 | 0.014 | 0.022 |
|  |  | 5 | 44 | 5 | 0.114 | 0.099 | 0.135 | 119 | 6 | 0.050 | 0.046 | 0.057 |
|  |  | 10 | 44 | 13 | 0.296 | 0.261 | 0.335 | 122 | 11 | 0.090 | 0.084 | 0.098 |
|  |  | 15 | 40 | 13 | 0.325 | 0.284 | 0.370 | 110 | 15 | 0.136 | 0.127 | 0.148 |
|  |  | 20 | 28 | 4 | 0.143 | 0.119 | 0.178 | 54 | 6 | 0.111 | 0.099 | 0.129 |

S.D.: standard deviation, EGD: Esophagogastroduodenoscopy, 95% CI: 95% Confidence Interval

**Supplementary table 6. Changes among different severity of atrophy and intestinal metaplasia according time-course**

|  | | | Open group (n=143) | | Closed group (n=4) | |
| --- | --- | --- | --- | --- | --- | --- |
| Score | | | Mean | S. D | Mean | S. D |
| Gastric cancer risk score | | |  |  |  |  |
|  | Time after first EGD | 0 | 5.10 | 1.13 | 2.04 | 1.80 |
|  |  | 5 | 3.47 | 1.02 | 1.42 | 1.29 |
|  |  | 10 | 3.29 | 0.86 | 1.43 | 1.14 |
|  |  | 15 | 3.24 | 0.92 | 1.37 | 1.09 |
|  |  | 20 | 3.19 | 0.83 | 1.40 | 1.13 |
| Atrophy score | | |  |  |  |  |
|  | Time after first EGD | 0 | 1.99 | 0.10 | 0.85 | 0.60 |
|  |  | 5 | 1.88 | 0.26 | 0.84 | 0.58 |
|  |  | 10 | 1.86 | 0.28 | 0.90 | 0.59 |
|  |  | 15 | 1.80 | 0.31 | 0.85 | 0.58 |
|  |  | 20 | 1.83 | 0.33 | 0.90 | 0.64 |
| Intestinal metaplasia score | | |  |  |  |  |
|  | Time after first EGD | 0 | 1.31 | 0.51 | 0.50 | 0.59 |
|  |  | 5 | 1.26 | 0.57 | 0.44 | 0.54 |
|  |  | 10 | 1.25 | 0.53 | 0.46 | 0.57 |
|  |  | 15 | 1.32 | 0.61 | 0.48 | 0.55 |
|  |  | 20 | 1.29 | 0.63 | 0.45 | 0.51 |

|  | | | IM group (n=143) | | Non IM group (n=4) | |
| --- | --- | --- | --- | --- | --- | --- |
| Score | | | Mean | S. D | Mean | S. D |
| Gastric cancer risk score | | |  |  |  |  |
|  | Time after first EGD | 0 | 4.65 | 1.41 | 0.58 | 0.83 |
|  |  | 5 | 3.12 | 1.21 | 0.65 | 0.94 |
|  |  | 10 | 2.99 | 1.03 | 0.67 | 0.78 |
|  |  | 15 | 2.97 | 1.00 | 0.48 | 0.60 |
|  |  | 20 | 2.86 | 1.01 | 0.46 | 0.62 |
| Atrophy score | | |  |  |  |  |
|  | Time after first EGD | 0 | 1.80 | 0.36 | 0.41 | 0.55 |
|  |  | 5 | 1.70 | 0.41 | 0.43 | 0.52 |
|  |  | 10 | 1.71 | 0.42 | 0.50 | 0.51 |
|  |  | 15 | 1.67 | 0.40 | 0.39 | 0.45 |
|  |  | 20 | 1.68 | 0.43 | 0.33 | 0.49 |
| Intestinal metaplasia score | | |  |  |  |  |
|  | Time after first EGD | 0 | 1.22 | 0.52 | 0.00 | 0.00 |
|  |  | 5 | 1.11 | 0.62 | 0.18 | 0.43 |
|  |  | 10 | 1.12 | 0.58 | 0.14 | 0.32 |
|  |  | 15 | 1.20 | 0.63 | 0.09 | 0.24 |
|  |  | 20 | 1.12 | 0.67 | 0.13 | 0.23 |

S.D.: standard deviation, EGD: Esophagogastroduodenoscopy
