# Supplementary figures and images for: Long-term endoscopic gastric mucosal changes up to 20 years after Helicobacter pylori eradication therapy
Source: Sci Rep. 2024 Jun 6;14:13003. doi: 10.1038/s41598-024-63928-6 (PMC11156848; doi:10.1038/s41598-024-63928-6)

Supplementary Figure 1

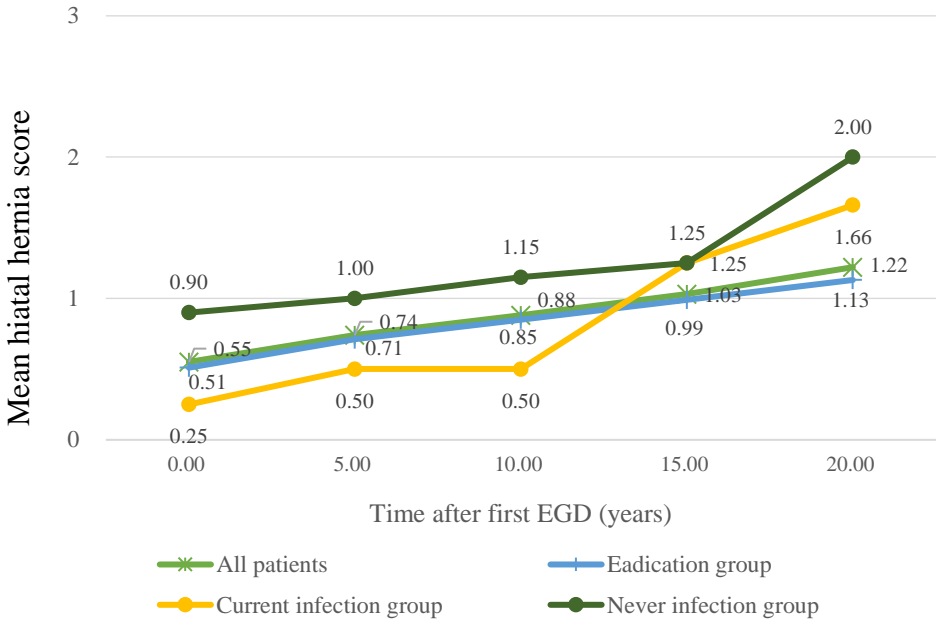

Supplementary Figure 2

\* : p<0.05 PH group vs non PH group

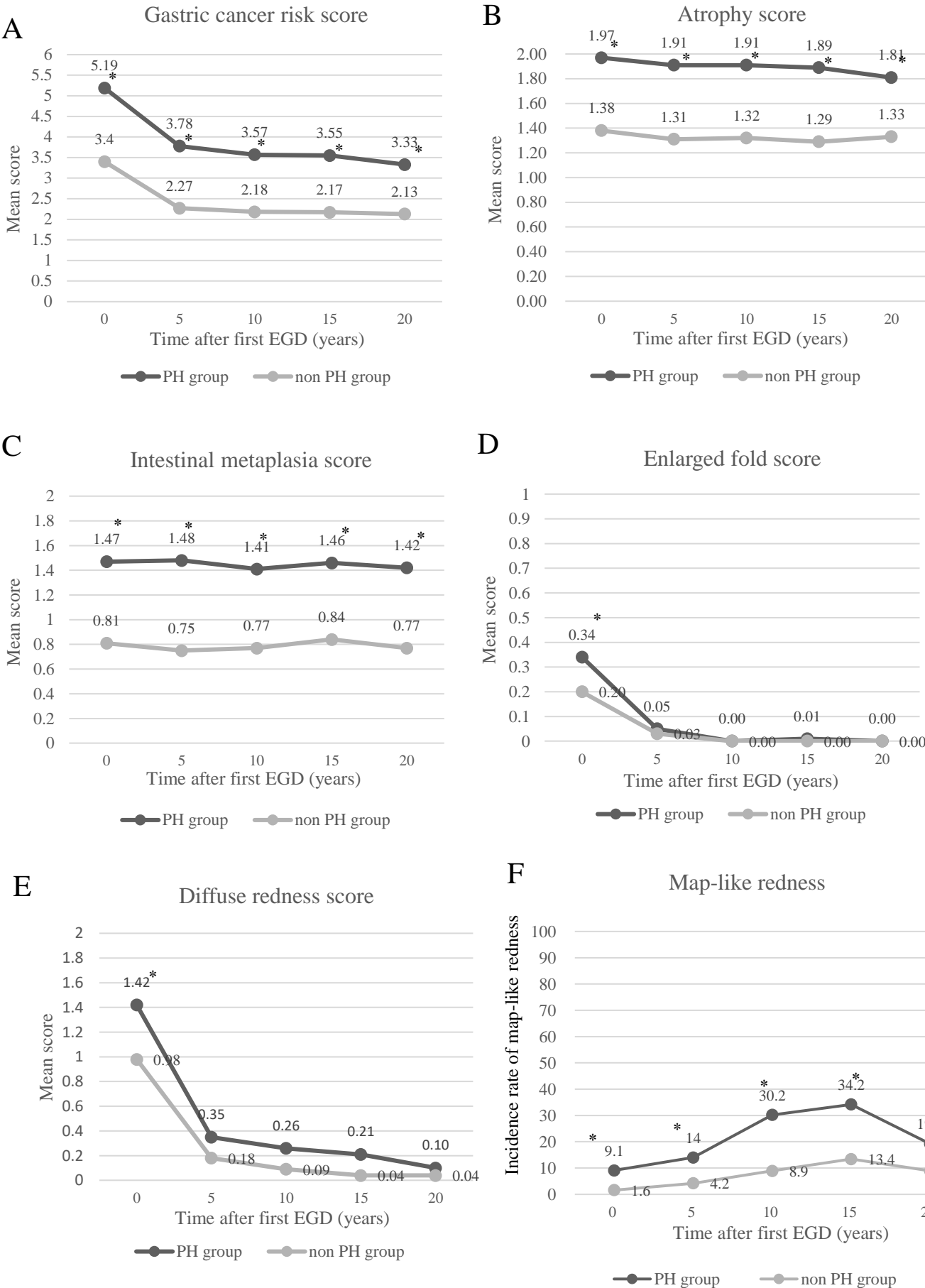

Supplement: Supplementary file 2 — Supplementary Figures. [file 41598_2024_63928_MOESM2_ESM.pdf]
